# Geosensor Data Representation Using Layered Slope Grids

**DOI:** 10.3390/s121217074

**Published:** 2012-12-12

**Authors:** Yongmi Lee, Young Jin Jung, Kwang Woo Nam, Silvia Nittel, Kate Beard, Keun Ho Ryu

**Affiliations:** 1 Database/Bioinformatics Lab, Chungbuk National University, Cheongju 361-763, Korea; E-Mails: ymlee@dblab.chungbuk.ac.kr (Y.L.); khryu@dblab.chungbuk.ac.kr (K.H.R.); 2 Korea Institute of Science Technology and Information, 245 Daehangno, Yuseong, Daejeon 305-806, Korea; 3 Department of Computer and Information Engineering, Kunsan National University, Kunsan 573-701, Korea; E-Mail: kwnam@kunsan.ac.kr; 4 School of Computing and Information Science, University of Maine, Orono, 5711 Boardman Hall, Rm. 344, Orono, ME 04467, USA; E-Mails: nittel@spatial.maine.edu (S.N.); beard@spatial.maine.edu (K.B.)

**Keywords:** sensor data abstraction, sensor data representation, geosensor network, slope grid, GIS, surface model

## Abstract

Environmental monitoring applications are designed for supplying derived and often integrated information by tracking and analyzing phenomena. To determine the condition of a target place, they employ a geosensor network to get the heterogeneous sensor data. To effectively handle a large volume of sensor data, applications need a data abstraction model, which supports the summarized data representation by encapsulating raw data. For faster data processing to answer a user’s queries with representative attributes of an abstracted model, we propose such a data abstraction model, the Layered Slopes in Grid for Sensor Data Abstraction (LSGSA), which is based on the SGSA. In a single grid-based layer for each sensor type, collected data is represented by slope directional vectors in two layered slopes, such as height and surface. To answer a user query in a central monitoring server, LSGSA is used to reduce the time needed to extract event features from raw sensor data as a preprocessing step for interpreting the observed data. The extracted features are used to understand the current data trends and the progress of a detected phenomenon without accessing raw sensor data.

## 1. Introduction

Geosensor networks are utilized in environmental monitoring applications to analyze natural phenomena [1,2]. Geosensor network data generally is voluminous, has streaming properties, and undergoes changes [3]. In sensor network applications, it is difficult to establish a high level context such as a global behavior, because the context needs to combine the numerous complex local actions of each node [4]. For example, we can understand changes of pollution status such as the change of a polluted area and a pollution level after getting all of sensor data for each local area. Deriving useful information from raw sensor data directly without any data representation is challenging because more time is needed to combine raw data. For example, some queries are difficult to answer, such as, “What is the dispersion speed of the detected air pollution?”, “Where is an area, where the air pollution level has changed frequently ?”, or “Where is an area, which shows steeply changed dust levels?”

**Figure 1.**
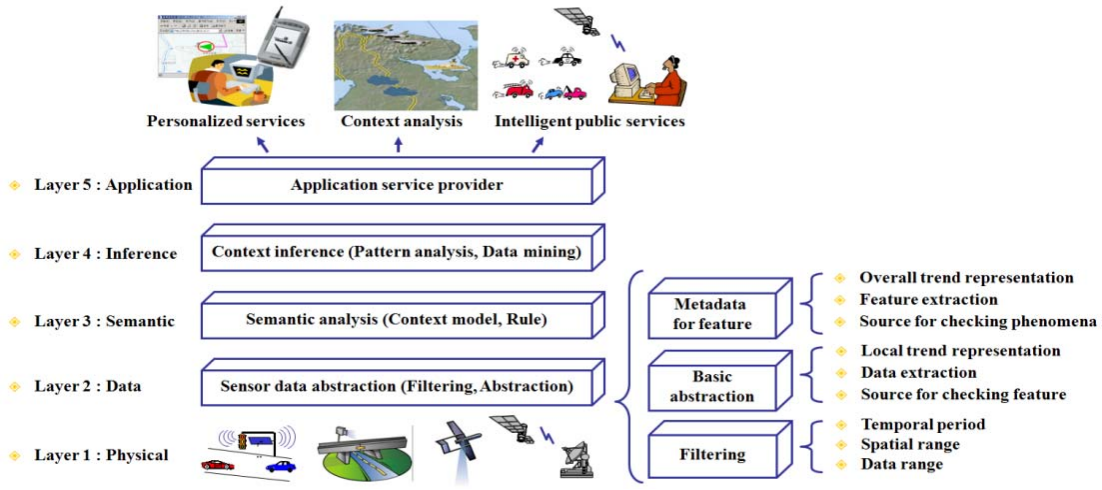
The data processing step in environmental monitoring system.

In an environmental monitoring application, the observed sensor data is used for understanding the current situation with data abstraction, semantic analysis, and context inference, as shown in the data processing steps of Figure 1 which are derived from context aware system [5,6]. An environmental monitoring application needs a well-organized data representation model to quickly interpret data and to extract the features of a detected phenomenon for processing user queries, because such an application must process large amounts of sensor data with the environmental information such as geospatial information. An additional layer, which provides logical data independence, is useful for handling large volumes of data by encapsulating the irregular data as a data management and interpretation step [7,8].

In this paper, Layered Slopes in Grid for Sensor Data Abstraction (LSGSA is designed as an additional layer to represent large volume of sensor data, to support faster data processing to quickly answer users’ queries, such as tracking air pollution areas. When a query is processed, LSGSA is used to reduce the time required to extract features from a large volume of sensor data by representing local sensor data in a centralized server. In LSGSA, a single grid-based layer presents received sensor data for each sensor type, such as temperature or humidity. In a layer, a surface and a height slope represent the collected sensor data by using direction vectors [9]. A surface slope represents the surface trend by collecting an overall direction for each cell, which is derived from the maximum values of subcells of a cell. A height slope shows an internal trend that is a set of height directions, which are derived from the difference between the maximum and minimum values of a cell.

Besides, a historical gradient coordinate is utilized for showing the historical data trend changes of the surface and height slopes. When a monitoring system builds a view or interprets data, this abstracted data is used for rapidly processing data as a basic data unit instead of raw sensor data.

## 2. Related Work

There is much interest in using network sensor systems for predicting and forecasting environmental events. There are various kinds of environmental monitoring applications such as CORIE for guiding vessel transportation and forecasting system [10], PODS for monitoring rare and endangered plants species [5], Short-term Inundation Forecasting for Tsunamis (SIFTS) in the NOAA tsunami warning center [11], Physical Oceanographic Real-Time System (PORTS) [12], Center for Coastal Margin Observation & Prediction (CMOP) [13], FloodNet for providing the functional floodplain conditions at a particular location [14], and a framework of *in-situ* sensor data processing systems, which area prototype of air pollution monitoring systems [6]. These environmental monitoring applications process large amounts of sensor data with the related environmental information such as a climate change, geological features, and ocean currents.

An abstraction, which makes a block of low level data, is used for economically processing queries or deriving a high level context in a sensor network or a sensor network application [4,8,15]. The architecture of MauveDB is proposed as an abstraction, which is classified as a model-based view [7,16]. This additional layer between the raw sensor data and an application view is used to filter raw data and to represent a summarized observation with an approximation model. This view covers missing data and removes any spatial and temporal biases in a system. When a system processes the set of queries, this view is useful to significantly improve a performance. Sensor Abstraction and Integration Layers (SAIL) [8,17], which is designed for context awareness, provides a layered architecture for simplifying the data acquisition and the node interaction between WSN applications and existing context-aware systems [8]. SAIL consists of three layers: Access, Abstraction, and Integration layers, which support WSN discovery, sensor controls, and event monitoring in an OSGi service platform [18]. These abstraction techniques are also used in sensor networks. Global Sensor Networks (GSN) employs virtual sensor abstraction, which is proposed to integrate sensor network data from various remote sensor data sources by using XML-based deployment descriptors and SQL queries [15]. This abstraction is useful to simply access the host of heterogeneous technologies by hiding arbitrary data sources with the virtual sensor abstraction with XML-based declarative deployment descriptions. An abstraction of regions [4] is designed for simplifying application design by encapsulating the details of low-level communication patterns, resource usage, data sharing, and collective operations in local regions of the network. In sensor network applications, it is difficult to establish a high level context such as a global behavior, because a lot of complex local actions for each node they need to be combined [4]. In order to reduce communication and data processing overheads, the abstract regions provide fairly low-level data representations such as building blocks for higher-level systems.

This data representation is also used to visualize sensor data such as Ocean of information [19], video visualizations [20], and Spatio-Temporal Knowledge Discovery [21]. In [19], Maritino described a visualization tool for real-time mobile activity using patch graphs, which show cell mobile activity density and the current number of calls with real satellite images. The patch graph of this paper also uses patches and the z coordinates of grids.

In most cases, frequent access to raw sensor data is required to answer a user’s queries. LSGSA is designed to reduce the time required to access raw sensor data to process a user’s queries by providing the representative attributes to present the detail surface and internal data trends with a slope grid.

## 3. Layered Slope Grid for Sensor Data Representation

To understand the detected phenomena, it is required to interpret data from transmitted sensor data with feature extraction. As a preprocessing step for analyzing a huge volume of sensor data, most environmental monitoring applications need a well-organized data representation model with rapid data processing which abstracts local data.

To design an abstraction model, inspiration can be derived from a spatial data access structure, such as TIN [22], or a grid [23]. These two major methods are effective for showing measured data or the conditions of a specific area such as a graph and a terrain surface model in GIS [24]. TIN is typically used to show a surface with high precision by describing the surface at different levels of resolution with the complex data structure in storage [25,26]. When a system must handle large volume of data, TIN is rarely used [25]. Grids are used to represent the terrain data in lots of large data providers such as the USGS, because a grid can store and manipulate large amounts of data with small storage size [24].

### 3.1. Layered Slope Grid Model

In this section, we describe Layered Slopes in Grid for Sensor Data Abstraction (LSGSA). Let *S =* {*s_1_*, *s_2_*, …, *s_i_*, …, *s_n_*} be a set of geosensors in a two-dimensional Euclidean plane. Geosensors is a tuple of (*sensor Id*, *sensor Type*, *x*, *y*), and a geosensor data is a tuple of (sensor id, value).

*Definition 1:Grid and Cell*. A grid G is a set of non-overlapped cells splitting a rectangle region, in other word G = {*g*_1_, *g*_2_, …, *g*_m_}. Also, each cell of a grid is a tuple of (cell-Id, min_x, min_y, min_z, max_x, max_y, max_z, start time, end time).

*Definition 2:LSGSA*. LSGSA is a set of LSGSA Cells, LSGSA = {*c*_1_, *c*_2_, …, *c*_m_ }. Each cell of LSGSA is a tuple of (cell-Id, surface-slope, height-slope, min_x, min_y, min_z, max_x, max_y, max_z, start time, end time).

∵ surface-slope = {1st DH, 2nd DH, 3rd DH, overall DH}
height-slope = {DH, min_value, max_value}
DH = {direction, height}

**Figure 2.**
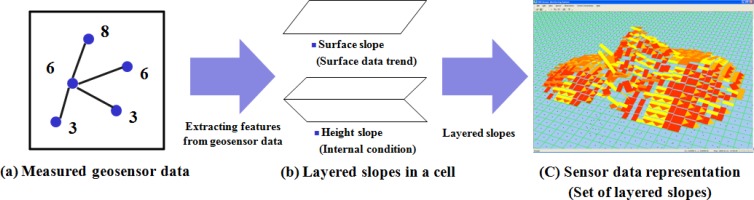
Sensor data representation with LSGSA.

Figure 2 shows a sensor data representation with LSGSA. LSGSA, which is based on a grid, is designed for faster data processing with data trend representation as one of preprocessing steps for analyzing current conditions and for answering users’ queries in a centralized monitoring application

LSGSA is used to represent the data trends for each sensor typeas a single grid-based layer such as LSGSA for temperature layer or the LSGSA for humidity layer.

**Figure 3.**
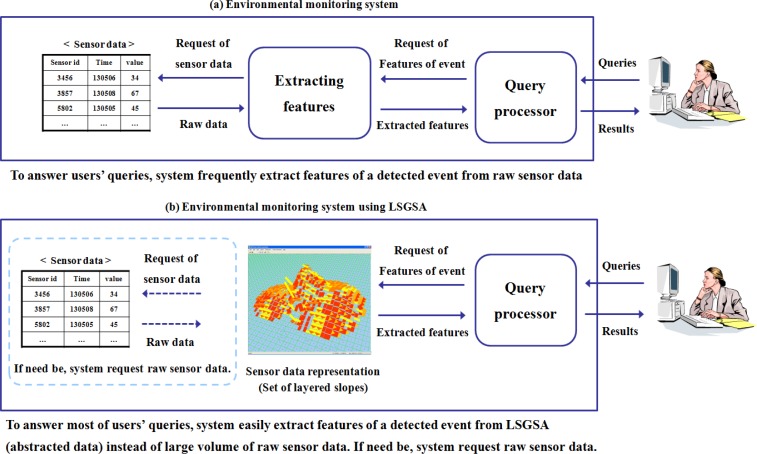
Query processing with LSGSA.

Figure 3 shows that LSGSA is useful for supporting faster data processing for users’ queries by utilizing the attributes of LSGSA. When a monitoring system (b) processes users’ queries, LSGSA is used to reduce the time needed to extract the features of the detected event such as the pollution area which has a continuously increased dust level, a pollution area which has a higher dust level than dust level 4, and the boundary of dust pollution. A system can easily extract the features of a detected event from LSGSA instead of the raw sensor data, because LSGSA already has the representative attributes of raw sensor data for each cell such as the overall direction of a surface slope, the vertical distribution of a surface slope, and the historical gradient of two slopes.

### 3.2. Surface and Height Slopes for Current Data Representation

**Figure 4.**
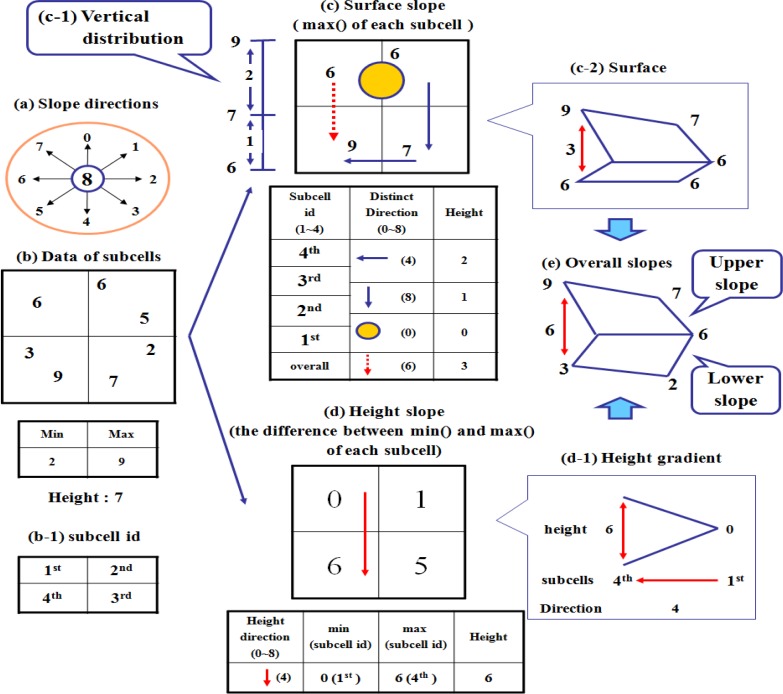
The layered slopes for data abstraction in a cell.

In LSGSA, the surface and height slopes are used to represent the current data trend as shown in Figure 4.

The surface slope represents the surface trend of a phenomenon for each sensor type such as a smooth or an irregular condition. Each cell of a surface slope has four direction vectors (three separate directions and overall direction), which are derived from the maximum values of four subcells of each cell. A height slope shows the internal trend of a phenomenon for each sensor type. Each cell of a height slope has a direction vector to present an internal condition within a cell, which is derived from the difference between the minimum and maximum values of a cell.

Two slopes are based on the tilted plane, which is used to show a surface area [26]. When showing a data trend, a tilted plane is better than a horizontal plane, because a tilted plane shows different data trends even though the heights of cells are the same. The slope direction vector [9] is used for describing the tilted plane of a cell by pointing to M subcell from m subcell after extracting m subcell, which has min() and M subcell, which has max().

When sensor data (b) is transmitted to a centralized server using an economical data acquisition method, LSGSA derives a surface and a height slopes to represent a current condition by defining an overall direction for each slope that is one of the predefined directions (a). For each cell, a surface slope (c) represents a surface data trend with max(), the maximum sensed value of each subcell (b-1). To make a surface slope, LSGSA sorts four max() of subcells and derives three separated directions to show a separated slope between two neighboring subcells in order of max() of subcells. A vertical distribution (c-1) shows the heights of three separate slopes. These separate directions are useful to identify parts of a detected event such as the fore, the middle, and the heel parts of a dust dispersion. Finally LSGSA derives an overall surface direction by combining these three separate slopes.

For each cell, a height slope (d) represents an internal condition with the difference between min() and max() of each subcell. To build a height slope, LSGSA defines an overall height direction to show the overall height gradient (d-1) between two subcells, which have minimum and maximum differences. For example, in Figure 4, the derived height direction (d-1) is 4, which indicates 4th subcell from the 1stsubcell and its height is 6, which shows the height gradient between the 1st and 4th subcells. This overall height gradient is used to understand the internal data trend of the detected phenomenon. For example, the height gradient of a height slope shows a suddenly changed condition such as an accident. With these surface and height slopes, LSGSA creates overall slopes (e), which describe overall data trends in a cell as a basic data abstraction of a local area by combining a surface direction and a height direction. Finally, LSGSA shows a global surface data trend by combining an overall data trend of a cell (local area).

**Figure 5.**
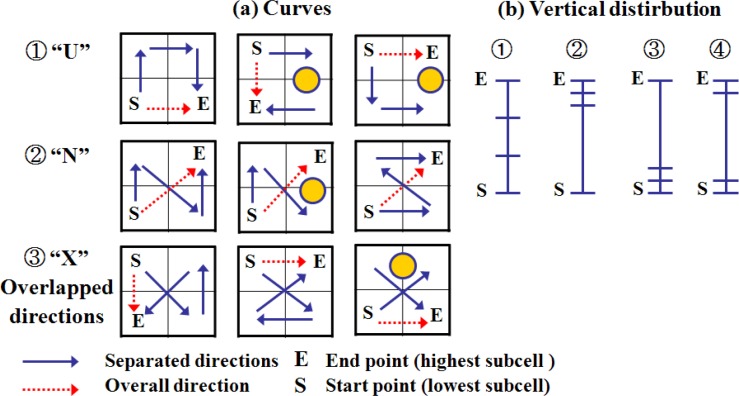
The relations of the separated directions of a surface slope.

The two slopes of LSGSA are also used to derive additional characteristics to simply understand a current data trend. For example, Figure 5 shows the relations of three separate directions of a surface slope such as a curve and a vertical distribution. In curves (a), three separate directions, blue arrows, are defined as one of predefined directions (direction: 0–7) or a dot (direction: 8, flat plane) through four subcells. The overall surface direction, a red dotted arrow, is defined from the start point (S) to the end point (E).When we link the sorted three separated directions, it derives a curve such as ①–“U”, ②–“N”, and ③–“X” as shown in a space-filling curve such as Peavo (π) and Hilbert curve [23]. This curve simply shows a surface condition. For example, both “U” and “N” curves show a simple surface gradient, which is similar to a spiral or zigzag stairs. The “X” curve shows an embossed surface condition. The vertical distribution (b) of a surface slope is derived from the heights of three separate slopes. This curve and a vertical distribution of a cell are used to understand current local surface conditions in detail.

### 3.3. A Historical Gradient Coordinate for Presenting Data Trend Change

**Figure 6.**
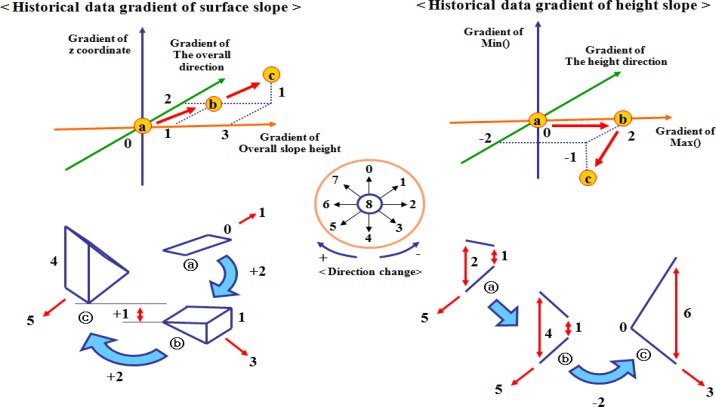
The historical gradient coordinate and data trend changes.

Figure 6 shows the history of the data trend change of the surface and the height slopes of a cell, which is described as the movement of a point in 3-dimensional space. Here, the movement means the difference between previous and current trends of the cell such as ⓐ, ⓑ, and ⓒ.

The historical gradient coordinate of a surface slope consists of three axes to present the gradients of the z coordinate, an overall direction, and a height of a surface slope. The ⓐ point (0, 0, 0) is moved to ⓑ point (1, 2, 0) by adding 1 unit of height and moving 2 directions. The direction, which is changed in a clockwise direction, is presented as a positive integer in the axis about the overall direction gradient. The direction is changed in the boundary [−4−+4]. If the direction is not changed or changed to 8, the gradient of the direction is set to 0. When a historical gradient coordinate is updated again, the ⓑ point is also moved to ⓒ point (3, 2, 1). From these movements, a user can obtain the information about the change of z coordinate, a height and a direction. The historical gradient coordinate ofa height slope consists of three axes to present the gradient of min(), the gradient of max(), the height direction of a height slope. Min() and max() can be changed even though the difference between them is the same over time.

**Figure 7.**
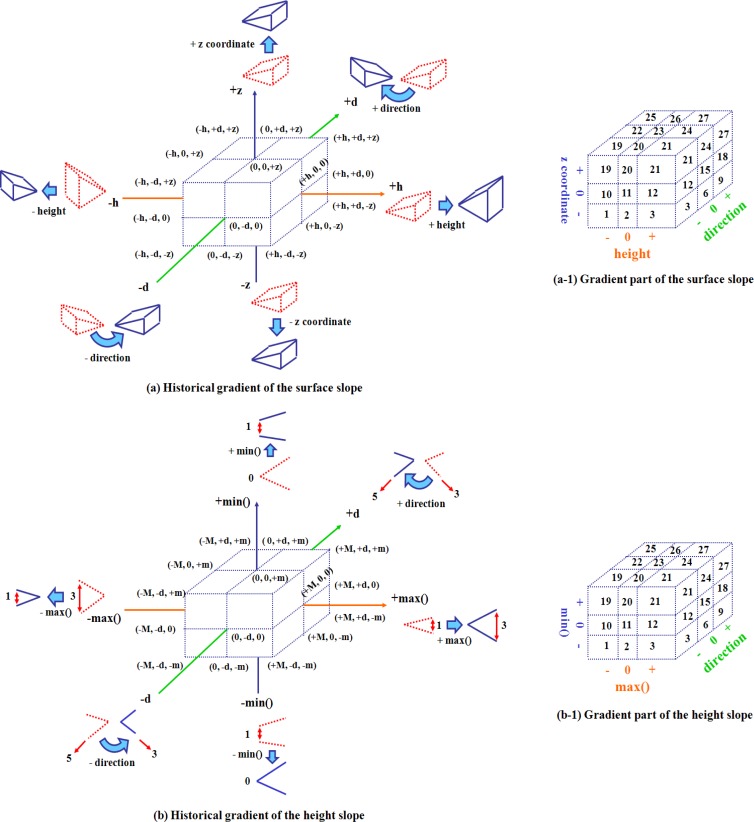
The historical gradient parts for the gradient count.

Figure 7 shows the historical data gradient of a surface and a height slopes such as (a) and (b). In these historical gradients, a position on the three dimensional coordinates shows a similar gradient style. For example, the points, which have positive integers on the height gradient axis of (a), indicate the increased heights, regardless of values. In (b), the points, which have negative integers on the gradient axis for min(), also show the decreased min()as many units have negative values.

To evaluate this gradient style of a cell, we define 27 historical gradient parts (a-1) and (b-1), which show similar gradient styles. First, we divide an axis into three parts such as (−, 0, +) and make the gradient parts by combining three axes. The cells included in the same gradient part show similar data trend changes, even though they have different gradient values. If the gradient part of a cell is changed, a user recognizes that the data trend of a cell is changed. Next, a gradient count is utilized to check the change of the historical gradient part of a cell. For example, when the data trend of a cell is updated, if the gradient part of a cell is changed, the cell’s gradient count is set to 0. If the gradient part is not changed, the cell’s gradient count increases. If a cell keeps a high gradient count for a long time, the condition of a cell, which is defined as a local area, is almost not changed. This gradient part is also utilized to search cells under similar conditions.

Depending on the application, the storage space is reduced by adding some points (historical gradient data). The trace of the points can be compressed by binding some points for a specific period. For example, the historical data gradient of a surface slope of Figure 6 shows the summarized movement (4, 4, 1) obtained by compressing ⓐ→ⓑ→ⓒ into ⓐ→ⓒ.

## 4. Data Representation Update

**Figure 8.**
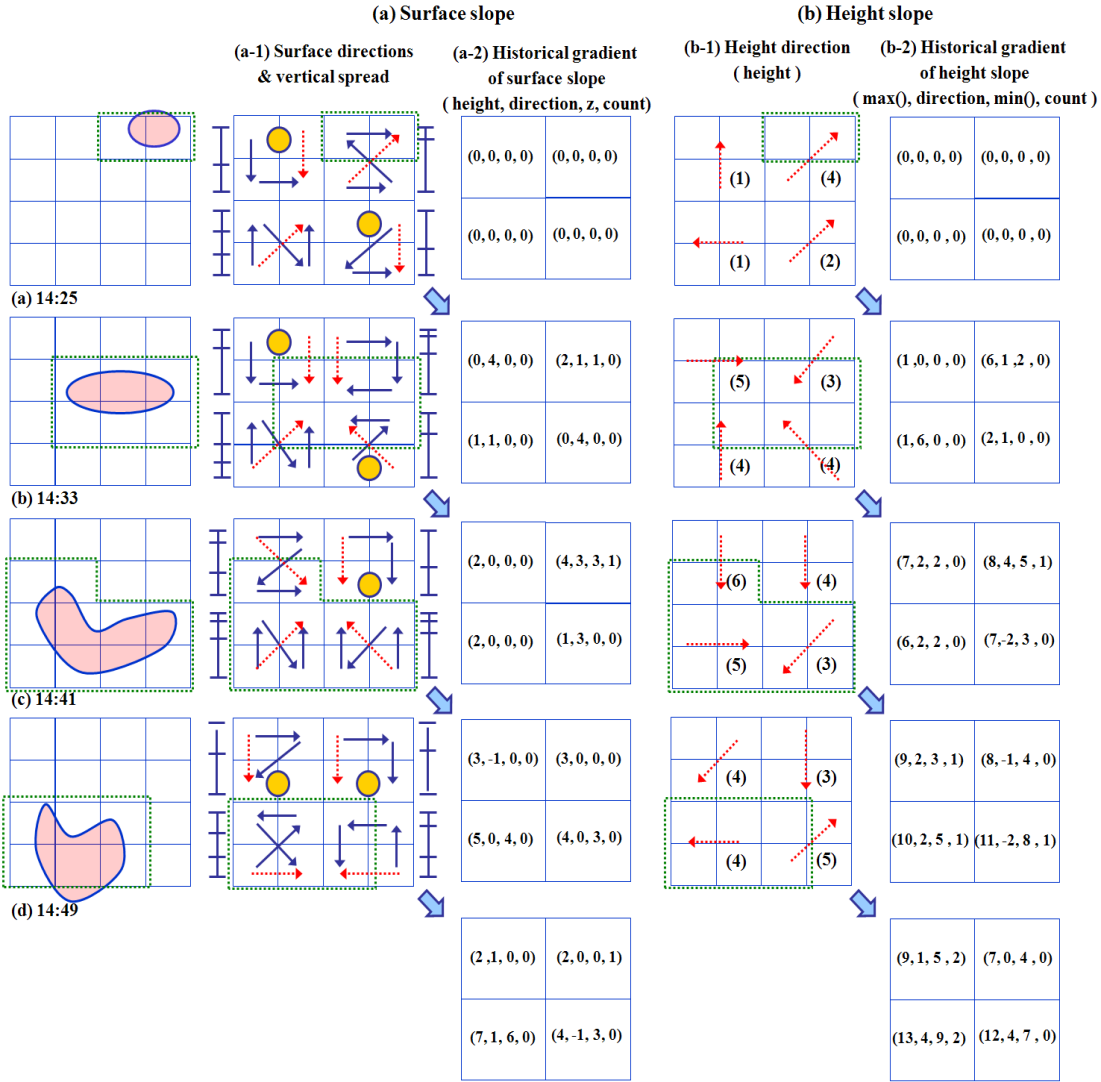
The representation update of the SGSA.

To show the change of some detected dust pollution, LSGSA updates its data representation over time in Figure 8. With the derived directions and their heights, the observed status is used for a monitoring system to understand the characteristics of an event. From this presentation, we can recognize the movement of a phenomenon from north to southwest by checking not only the change of the surface and the height slopes but also the historical gradient data. If an event has a higher temperature than a normal condition, the slope directions of temperature LSGSA generally indicate the center of the event, because a slope direction points to the subcells having a high value of a cell.

A height direction generally indicates a rapidly changed condition under an event, because a height slope shows the difference gradient between min and max subcells of a cell as shown in Figure 4. The detected dust in a particular area is searched by following the directions of a surface or a height slopes. For example, in our implementation, this increased height is distributed around the boundary and the heel of the event, as shown in Section 6.

LSGSA shows the current data trends over time, which are summarized from both synchronized and unsynchronized sensor data. Each cell has the time period attributes such as start time and end time to keep sensor data for the predefined time interval such as 10 min from the sensor data arrival time. When new sensor data is transmitted in this time period, the attributes of each cell are updated and the time period is also updated.

## 5. Data Processing with LSGSA

**Figure 9.**
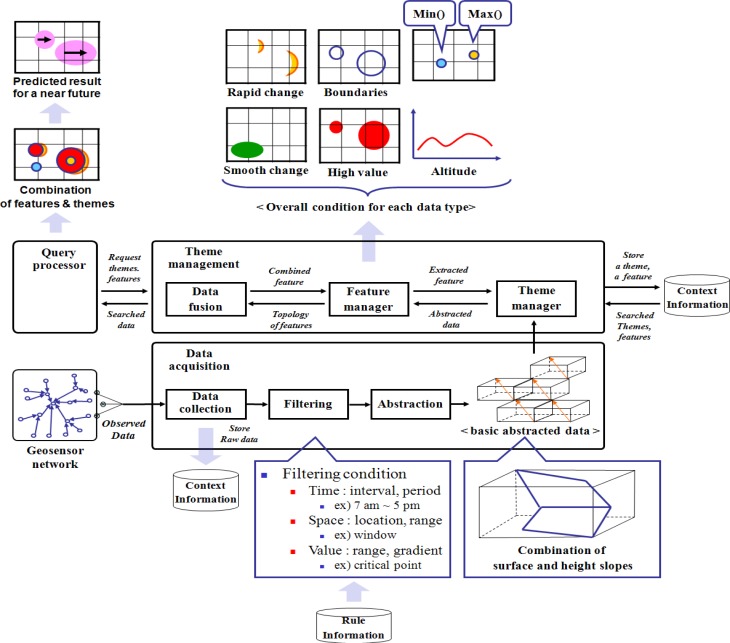
The data processing with abstracted sensor data.

To answer a user query to find a particular area, which will be polluted by dust in the near future, a system extracts some features of the detected dust, such as a boundary and a rapidly or smoothly changed area in Figure 9. The current dust pollution area is extracted by searching for cells, which have a high dust value over a critical point of the dust data abstraction layer. The boundary of the pollution area is also determined by checking for a steep surface and an increased height with low z coordinates.

## 6. Representation of Detected Events with Geosensor Data

**Figure 10.**
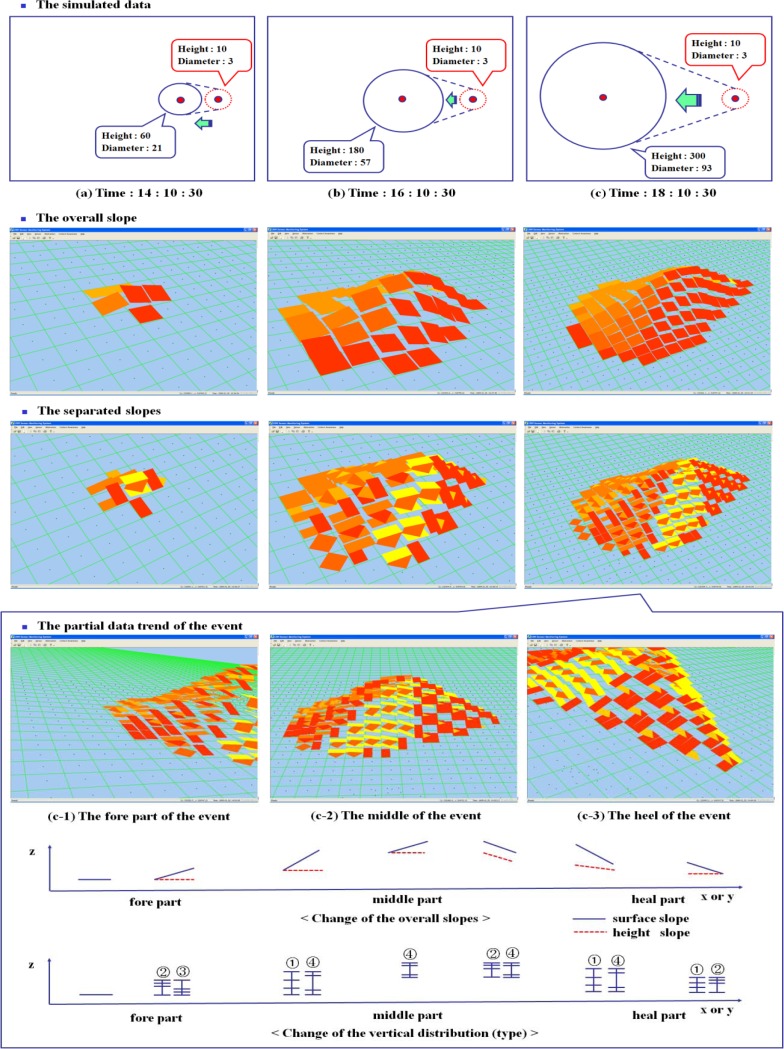
Tracking the simulated phenomenon with LSGSA.

The slowly or rapidly changed conditions in the pollution are found by extracting smooth or irregular surface slopes.Finally, the system combines the extracted features and derives a global pollution progress direction and a speed, as shown in Figure 10. If a user wants to get a detailed data representation for a particular area such as TIN [22], a centralized system subsequently constructs a TIN-based model with the original sensor data in the cells after searching the cells included in a particular area with LSGSA

The designed LSGSA is implemented to represent sensor data and to extract features of a detected phenomenon to answer users’ queries. We assume that all of the detected data are transmitted to the centralized server for intensive data analysis such as the pollution prediction in near future. The implemented LSGSA utilizes 60,000 simulated static sensors and 10,000 cells. The simulated sensor data is continuously represented over time with the surface and height slopes of LSGSA.

Figure 10 shows an event movement tracking with LSGSA, which includes the extracted features for three parts of an event such as the change of overall slopes and vertical distributions. To show the attributes of LSGSA, a sensor data generator is used to simulate a moving event under the assumption that all of sensor data is synchronized. When an event is moved, the data values of the cells included in the event are increased by 10 units per every sampling point (10 min). The values of other cells, which are not included in the event, decrease to present the condition change, which comes back to a normal condition. LSGSA is used to find the event area and its data level by searching the overall slopes, which are included under abnormal condition. The separated slopes (c) are also used to get the characteristics of each part of the event, because they show a detailed condition. The fore part (c-1) of the event shows a low height and low z coordinate. Most of the slope directions of these cells point in the same direction. These directions indicate the fact that an event is coming. In the middle part (c-2), a system recognizes that the condition of this area is rapidly changed, because their height and z coordinate are quickly increased. The values of attributes of a cell are steeply changed but the surface and the height directions are not changed until the center of the event is passed. In the heel part (c-3), slopes present the decreased height and z coordinate, which come back to normal conditions after the center of the event is passed. With these characteristics of each part, a system can extract the specific cell for data analysis by implementing user defined rules such as surface height ≥4 and gradient count >10.

**Figure 11.**
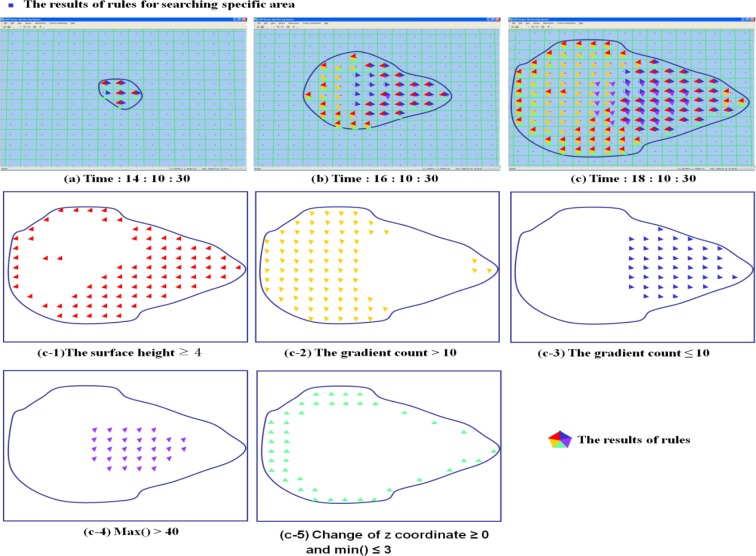
The results of user defined rules for checking the characters of the detected event.

LSGSA is used to find the various features of the event without accessing raw sensor data. To check the partial conditions of the event, five rules are used to search the cells under an interesting condition in Figure 11. The results of the rules are shown with the different colored triangles, which form the parts of a pentagon. Rule (c-1) is used to find a rapidly changed condition by searching the height in a height slope ≥4. The results (red) are distributed around the boundary and the opposite side of the progress of the event. Rule (c-2) searches the area, which keeps its surface direction for a while (100 min) by checking the surface gradient count >10. The results (yellow) spread around the fore and middle part of the event except for the cells around the summit, which shows maximum dust levels. This high gradient count means that the selected areas were under a similar condition for a minimum of 100 min. Rule (c-3) is used to find the areas in which conditions are changed in the event. After the center of the event is passed, the selected cells (blue) appear. To get the cells around the summit, rule (c-4) checks the max() of cells. Rule (5) finds the boundary of the event by checking the change of z coordinate and min() of a cell. It is also required to combine the selected cells in order to find the results of rules in answering requests such as: “Track the condition change of cells, which were under a similar condition or in a specific area”, and “Where is an area in which conditions frequently changed in a detected event?”

**Figure 12.**
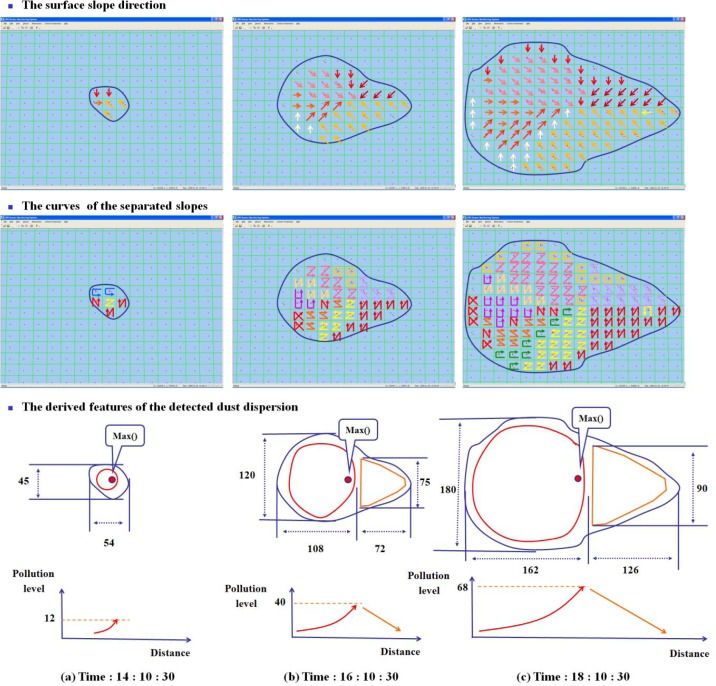
The curves of LSGSA and the extracted features of the detection.

The attributes of LSGSA are useful to understand the conditions of the detected event, because they are used to derive the characteristics of an event such as the dispersion speed and the current polluted areas with varied pollution levels. The curves of the LSGSA present different conditions depending on their arrangement, even though their slope overall directions are the same. Their vertical distributions also show the varied conditions. In Figure 12, a current pollution area has an increasing pollution part (fore part) and a decreasing pollution part (heel part). Some areas, red circles, have\an increasing pollution level, because this area is still affected by dust, which is continuously dispersed from a source in the area. A system extracts some features of the pollution from LSGSA. For example, the changed extent of this area is (a) 1,620 m^2^→(b) 9,450 m^2^→(c) 24,840 m^2^. The detected pollution level is increased by 0.23 mg/min. The pollution level of the source is generally much higher than these detected values, which present the pollution level on the ground. The pollution level is frequently changed depending on the changed position of the source.

The system also derives from LSGSA that the dust pollution’s moving speed is 0.9 m/min. When the pollution source is moving, some places escape from the effects of the detected pollution. Other pollution areas, yellow line, show a decreased pollution level. This area will be back to normal conditions, because this area is beyond the current pollution affected area. The extent of change of this area is (b) 2,240 m^2^→(c) 9,180 m^2^. The detected pollution level is decreased by 0.14 mg/min.

## 7. Conclusions

In order to understand the characteristics of a detected event, an environmental monitoring application extracts and combines the interesting features such as current pollution area and its pollution level, and a pollution dispersion speed. An abstraction model is required to effectively interpret the conditions of a specific area by encapsulating sensor data. LSGSA is designed to quickly extract features from large volumes of sensor data by representing local sensor data with a surface and a height slopes. Besides, the historical gradient coordinate is also designed to check the historical data trend changes of a cell. When a monitoring system builds a view or interprets data, this abstracted data is used to rapidly process data as a basic data unit instead of raw sensor data. In the future, LSGSA will be combined with economical communication methods such as data aggregation or abstraction.

## Appendix Geosensor Data Update Algorithm of LSGSA

To explain the sensor data insertion and the data abstraction in LSGSA, Algorithms 1 and 2 show the data processing in cells of LSGSA. Whenever sensor data is received, the sensor’s location and its value are inserted into the cell of LSGSA as shown in Algorithm 1. The LSGSA updates its data representation with this updated cell over time.

**Algorithm 1.** Insert geosensor data to LSGSA.

**Algorithm LSGSA::insert_DataToLSGSA (GeosensorValues *geosensor)**
**input**: geosensor // the geosensor id
**method** :
//Whenever sensor data is received, the value is inserted into the cell, which includes the sensor’s location.
cell = get_CellId(geosensor.x, geosensor.y);
insert_DataToCell(cell, geosensor.value); // insert the geosensor value to the cell
**end**

**Algorithm 2.** Get surface and height slope’s directions of a cell of LSGSA.

**Algorithm LSGSACell::GetDirectionOfCell (LSGSACell *cell)**
**input** : cell // a cell of LSGSA
**output** : cell // the cell, which has surface and height slopes’ directions
**method** :
// a surface slope considers only max() of subcells.
cell.SurfaceSubcells[] = sort(getSurfaceSubcells(cell)); // In surface slope, each surface subcell has only max() of the subcell
cell.SurfaceMinSubcell = getSurfaceMinSubcell(cell.SurfaceSubcells[minimum]); // get a subcell which has minimum max().
cell.SurfaceMaxSubcell = getSurfaceMaxSubcell(cell.SurfaceSubcells[maximum]); // get a subcell which has maximum max().
**if**(cell.SurfaceMinSubcell.id = cell.SurfaceMaxSubcell.id){
// if there is only a subcell, surface slope is flat
cell.surface_direction = 8;
}
**else**{ // there are more two subcells.
// if max() of subcells are equal, the surface slope is flat.
**if**(cell.SurfaceMinSubcell.max() = cell. SurfaceMaxSubcell.max()){
cell.surface_direction = 8;
}
**else**{ // if max() of subcells are different, the surface is not flat.
// surface direction indicates SurfaceMaxCell from SurfaceMinCell
cell.surface_direction = GetSurfaceDirection(cell.SurfaceMinSubcell, cell.SurfaceMaxSubcell);
}
}
cell = getSeparatedDirections(cell.SurfaceSubcells[]); // get the order of surface subcells and their separated directions.
// a height slope considers only the difference between max() and min() of subcells
// each height subcell has only the difference between max() and min() of subcells
cell.HeightSubcells[] = sort(getHeightSubcells(cell));
cell.HeightMinSubcell = getHeightMinSubcell(cell.HeightSubcells[minimum]); // get a subcell which has minimum difference.
cell.HeightMaxSubcell = getHeightMaxSubcell(cell.HeightSubcells[maximum]); // get a subcell which has maximum difference.
**if**(cell.HeightMinSubcell.id = cell.HeightMaxSubcell.id){
// if there is only a subcell, its height slope is flat
cell.height_direction = 8;
}
**else**{ // there are more two subcells.
**if**(cell.HeightMinSubcell.difference = cell.HeightMaxSubcell.difference){ // if differences of subcells are equal
cell.height_direction = 8; // height slope is flat.
}
**else**{ // if differences of subcells are different, the height slope is not flat.
// height direction indicates HeightMaxSubcell from HeightMinSubcell
cell.height_direction = GetHeightDirection(cell.HeightMinSubcell, cell.HeightMaxSubcell);
}
}
**return** cell // the cell, which has surface and height slopes’ directions
**end**

The surface and height slopes of LSGSA are created to represent the updated sensor data for every predefined LSGSA update time. To get the direction of surface and height slopes, the Get Direction function of SGSA [9] is used in Get Height Direction and Get Surface Direction. Get Direction finds the overall direction of a slope from Surface Min Subcell to Surface Max Subcell with the predefined direction value and subcell ids, as shown in Figure 4. The time complexity of Get Direction to define the direction of a cell, which is similar to the search process of a tree, is log 8, because there are nine direction values such as eight directions (0–7) and flat (8). Get Direction is also called to derive each separate direction.
