# Correction: Lee, Y. *et al*. Geosensor Data Representation Using Layered Slope Grids. *Sensors* 2012, *12*, 17074-17093

**DOI:** 10.3390/s130405404

**Published:** 2013-04-22

**Authors:** Yongmi Lee, Young Jin Jung, Kwang Woo Nam, Silvia Nittel, Kate Beard, Keun Ho Ryu

**Affiliations:** 1 Database/Bioinformatics Lab, Chungbuk National University, Cheongju 361-763, Korea; E-Mails: ymlee@dblab.chungbuk.ac.kr (Y.L.); khryu@dblab.chungbuk.ac.kr (K.H.R.); 2 Korea Institute of Science Technology and Information, 245 Daehangno, Yuseong, Daejeon 305-806, Korea; 3 Department of Computer and Information Engineering, Kunsan National University, Kunsan 573-701, Korea; E-Mail: kwnam@kunsan.ac.kr; 4 School of Computing and Information Science, University of Maine, Orono, 5711 Boardman Hall, Rm. 344, Orono, ME 04467, USA; E-Mails: nittel@spatial.maine.edu (S.N.); beard@spatial.maine.edu (K.B.)

There are four mistakes at the table derived from the (c) surface slope of Figure 4 in [1]. The direction numbers are derived according to (a) slope directions. The overall direction number should be changed from 6 to 4. The distinct direction number between the 1st and 2nd subcells should be changed from 0 to 8. The distinct direction number between the 2nd and 3rd subcells should be changed from 8 to 4. The distinct direction number between the 3rd and 4th subcells should be changed from 4 to 6. The authors would like to apologize for any inconvenience this may have caused to the readers of this journal.

The new figure is provided below:

## Figures and Tables

**Figure 4.**
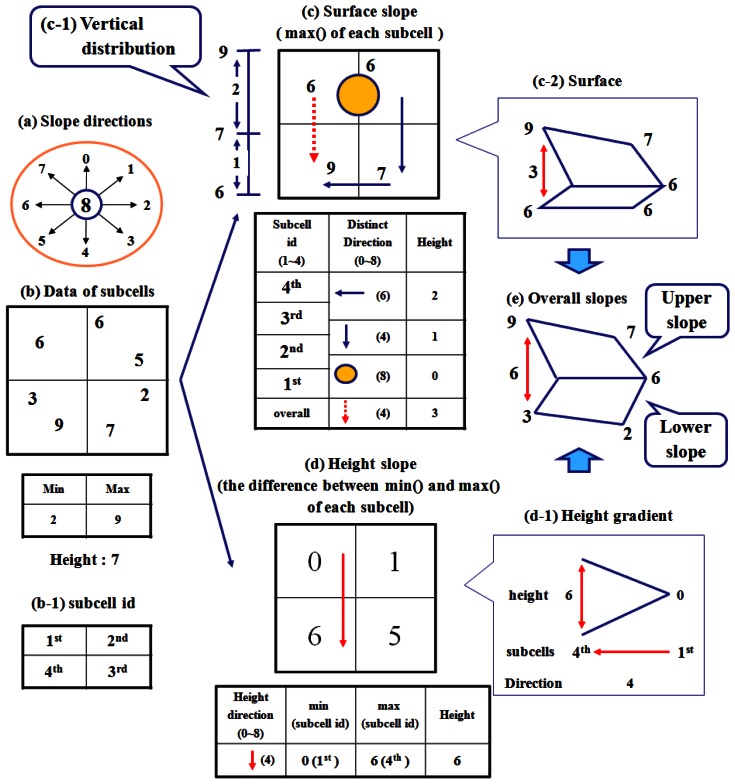
The layered slopes for data abstraction in a cell.
